# Influence of parental beliefs on parental support for young athletes

**DOI:** 10.3389/fspor.2026.1912532

**Published:** 2026-08-27

**Authors:** María del Pilar Méndez Sánchez, Rafael Peñaloza Gómez, Mirna García Méndez, Gabriela Carolina Valencia Chávez, Nancy Ponce Carbajal

**Affiliations:** 1 Facultad de Estudios Superiores Zaragoza, Universidad Nacional Autónoma de México, Mexico City, Mexico; 2 Facultad de Organizacion Deportiva, Universidad Autonoma de Nuevo Leon, San Nicolás de los Garza, Mexico

**Keywords:** barriers to sport success, emotional understanding, material resource support, parental disinterest, parental expectations

## Abstract

**Introduction:**

Parental influence is a central element in sport development. Therefore, parental beliefs and attitudes toward sport may influence the support provided to enhance the sport development of sons and daughters.

**Objective:**

The present study analyzed the relationship between parental beliefs and the support provided by parents to young athletes. Before this analysis, validity evidence was examined for the instruments used in the study.

**Method:**

A non-probability sample of 505 adolescents and young adults from eastern Mexico City was included, with a mean age of 20.6 years (SD = 2.85). All participants practiced either an individual or team sport.

**Results:**

Validity evidence was obtained for two measurement instruments. The Parental Beliefs about Sport Potential Scale was composed of four factors, and the Parental Support in Sport Scale was composed of three factors. Both scales showed adequate psychometric properties. In addition, significant positive and negative relationships were found between parental beliefs about sport potential and parental support.

**Conclusions:**

The results highlight the importance of parental influence in sport and suggest the need to promote forms of support that contribute to sport maintenance, participation, and performance.

## Introduction

Sport participation represents a specific form of physical activity rather than an interchangeable concept. Physical activity encompasses any bodily movement produced by skeletal muscles that requires energy expenditure (1), whereas sport is a subset of physical activity generally involving rules, formal practice, and competition (2, 3). In the present study, sport involvement refers to sustained participation in an individual or team sport, including at least one consecutive year of practice and experience in at least one sport competition. Beyond its physical demands, sport participation takes place within a social context in which family members, coaches, and teammates influence athletes' experiences, motivation, and continuity.

The family constitutes the first context of contact and formation for sport participation (4). As the primary agent of socialization, the family provides children with norms, values, and behavioral models (5) that contribute to athlete development. Research on physical activity, as the broader category within which sport participation is situated, has identified two types of parental strategies: direct strategies, such as taking children to places where they can be active, and indirect strategies, such as modeling physical activity and providing verbal encouragement (6).

Parental influence has an impact on the attainment of sport achievements and on the decision to continue practicing a sport or to abandon it. Previous studies have reported that parental support increases resilience, whereas conditional praise and high demands contribute to anxiety and reduce motivation (7). Parental support has also been identified as the main driver of intentions to continue participating in sport (8). It should also be considered that coaches and teammates are socialization agents who influence how the sport environment is perceived (9). However, perceived parental support throughout the process of accompaniment and involvement in sport activities allows athletes to develop commitment (10). Likewise, factors related to communication, the family environment, and involvement in the sport life of athletes play a relevant role (11). A favorable family environment is associated with higher physical self-concept in children and a higher frequency of physical and sport activity (12). Mothers and fathers who value sport practice are aware of its impact on health and on the development of social relationships in their sons and daughters; therefore, they become involved, provide support, and value the healthy lifestyle that sport contributes to their children, even though these activities may affect their lives and routines due to the time and effort required (13).

Parents play an important role in the lives of athletes at early ages, as they are responsible for guiding and supervising them. Likewise, parental support has a positive influence on achievement motivation and sport performance (14). Family socialization that prioritizes sport and physical activities is associated with greater motivation toward sport practice, directly influencing self-esteem and academic performance (15).

Parental influence is also notable in the achievement orientation of sons and daughters, since task orientation has been found to be explained by parental involvement in training and support during play. This encourages children to value mastery of the discipline and contributes to positive self-esteem and greater enjoyment of sport (16). Thus, parents who are involved in both education and sport tend to have children with recognized sport performance and academic achievement, which shows the positive role of parental participation in sport and education (17). Although greater parental involvement has been reported in team sports compared with individual sports (18), when athletes perceive low parental involvement, their performance and commitment to sport activities tend to decrease (19).

Similarly, family sport heritage represents an important element for sport continuity. The family plays a role in sport activities because family members have served as models and have supported and encouraged participation; these influential relatives are usually the father, mother, siblings, or cousins (20). Family quality of life also has a positive influence on sport performance, as athletes from families with better economic conditions and positive family interactions tend to achieve sport success (21). Undoubtedly, the provision of financial resources by the family for sport participation indicates that families make substantial investments in sport careers, although some may expect a certain return on that investment (9).

Parental beliefs that influence sport potential also develop within the family environment. Parental opinions about the abilities of sons and daughters influence how young athletes perceive their own skills; that is, when young people perceive positive beliefs about their abilities, they use internal criteria to evaluate themselves and accept greater challenges (13). Perceptions about the abilities of sons and daughters are parental expectations that have been understood as types of beliefs linking different domains of performance. These beliefs are influenced by parental experiences and attributes and affect the behavior of sons and daughters; in turn, they are also shaped by individual characteristics, such as sex, health status, and learning ability (22). Parental opinions about the sport abilities of sons and daughters influence their perceptions of their own skills and the development of their interests; in other words, if athletes perceive positive beliefs about their abilities from their mothers and fathers, they evaluate themselves using internal criteria and accept greater challenges (13). When parents show higher levels of perfectionism in sport, their children tend to follow similar patterns to those of their parents (19).

However, negative family behaviors may also occur, such as pressure, high demands, and an excessive emphasis on victory and performance, which create unrealistic expectations among athletes and, consequently, dissatisfaction with sport practice (9). Although most parents expect their sons and daughters to continue participating in sport, especially because of its health benefits, in many cases these expectations are not related to performance or competitive goals. This may occur either because parents do not trust the abilities of their sons and daughters, or because they seek to protect them from the frustration of not achieving such goals (13).

Parental involvement in youth sport is not always perceived in the same way by parents and athletes. Lienhart and Nicaise (23) found only modest agreement between their reports: compared with athletes, parents reported less directive behavior and pressure, but greater active involvement and praise and understanding. These discrepancies also varied according to gender and were generally smaller in mother–athlete than in father–athlete reports. Similarly, Lopes et al. (24) identified differences in perceptions of competition attendance, technical influence, and sport expectations among youth basketball athletes and their parents. Parents reported greater attendance at competitions, whereas athletes perceived greater technical influence and expectations. Earlier evidence also showed that parents rated the pressure they exerted as lower than their children did, while children's perceptions of paternal pressure were associated with less favorable feelings toward their sport (25). Qualitative research with parent–athlete dyads further indicates that behaviors intended as supportive, such as attending practices or providing performance feedback, may be experienced by athletes as controlling or overwhelming when they do not correspond with their preferences (26). Therefore, parental involvement cannot be understood solely through parents' intentions, as athletes' interpretations determine how support and pressure are experienced in the sport context.

Likewise, promoting gender equality in sport from the family context is crucial. Encouraging the participation of girls and women in sport activities contributes to breaking gender stereotypes and promoting a more inclusive and equitable sport environment. This not only benefits female athletes, but also enriches family dynamics and strengthens the values of equality and respect (27). One of the most marked beliefs in sport involves social perceptions of gender. Different sports are associated with gender; for example, sports such as football continue to be strongly associated with masculine identity, as do combat sports such as boxing or sumo, which significantly influences participation (28). Gender discrimination in sport, resulting from certain stereotypes associated with specific disciplines, creates obstacles for women that their male peers do not experience (29).

Other beliefs linked to physical activity and sport are related to barriers involving time, costs, transportation, accessibility, and opportunities (30, 31). Economic factors hinder sport trajectories, since much of the funding comes from athletes themselves and from family income. In most cases, athletes do not receive government funding, and maintaining a dual career requires strenuous efforts from the athlete (29).

Several instruments with validity evidence have been identified to evaluate perceptions related to parental beliefs and parental support. The Youth Sport Parental Support Questionnaire (YSPS-Q) includes four dimensions: autonomy support, emotional support, instrumental support, and informational support, with Cronbach alpha reliability values above.70 (32). The Parental Involvement in Sport Scale (EIPD, Spanish acronym), developed in Buenos Aires, Argentina, was designed for boys and girls between 9 and 15 years of age. It uses a Likert-type scale from 1 (Always) to 5 (Never) and includes three factors: Directive Behavior (*α* = 0.929), Support and Understanding (*α* = 0.871), and Active Involvement (*α* = 0.841) (33). Another instrument is the Parental Promotion of Physical Activity Scale (ACTS-MG), developed in Mérida, Mexico, which includes four response options ranging from strongly disagree to strongly agree. Its psychometric properties explained 45.54% of the total variance, and its dimensions are Inclusion in Structured Activities (*α* = 0.792), Logistic Support (*α* = 0.753), Restriction of Sedentary Activities (*α* = 0.729), and Modeling (*α* = 0.775). This instrument is aimed at parents or primary caregivers of children between 6 and 12 years of age who practice sport (34). The Parental Support Scale for Sport Training in Children (PSCSTS), developed in China, uses a 5-point Likert scale ranging from strongly disagree to strongly agree and explains 55.65% of the total variance. It includes the dimensions of policy support, communication support, family support, intrinsic motivation, school support, media support, and training quality support. The informants were parents whose children participated in sport training (35).

Although previous research has examined parental support and involvement in sport, less is known about the beliefs that athletes attribute to their parents regarding their sport potential and how these beliefs are associated with the different forms of support they perceive. Moreover, the available instruments have primarily focused on parental support, involvement, or the promotion of physical activity, whereas validity evidence for measures specifically addressing parental beliefs about sport potential in Mexican athletes remains limited. Accordingly, the present study aimed to analyze how parental beliefs influence the support provided by parents to young athletes. For this purpose, a cross-sectional instrumental design was used, since the first aim was to examine the psychometric properties of the instruments. Subsequently, correlational analyses were conducted to address the substantive research objective.

## Method

### Participants

The study included 505 adolescents and young adults with a mean age of 20.6 years (SD = 2.85), from eastern Mexico City, Mexico. Participants were recruited through secondary, upper-secondary, and higher education institutions, as well as through local sport networks and athlete groups in this area. The sample was selected through non-probability sampling. Participants met the following inclusion criteria: practicing an individual or team sport, having practiced that sport for at least one consecutive year, having participated in at least one sport competition, and being between 12 and 25 years of age. Cohabitation with parental figures was not required, as the instruments assessed participants' perceptions of parental beliefs and support regardless of household composition.

In the present study, the term young athletes was used operationally to encompass adolescents and young adults between 12 and 25 years of age who met the sport participation criteria described above. A total of 587 responses were initially obtained, of these, 82 were excluded because they did not meet one or more of the inclusion criteria, resulting in the final sample. Table 1 presents detailed sociodemographic information of the participants.

**Table 1:** Demographic data of the participants.

Variable	Frecuency	Percentage
Sex		
Male	293	58
Female	212	42
Family structure		
Mother, father and siblings	256	50.7
Mother and siblings	67	13.3
Mother and father	55	10.9
Mother only	50	9.9
Extended family	27	5.3
Father and siblings	11	2.2
Living alone	22	4.4
Other	9	1.8
Grandparents	8	1.6
Educational level		
Undergraduate degree	334	66.1
High school	144	28.5
Technical education	19	3.8
Postgraduate degree	8	1.6
Do your parents engage in physical activity or sport?		
No	273	54.1
Yes	232	45.9
	Mean	SD
Age at wich participants began practicing sport	11.46	5.46
Hours of training per week	9.20	5.26
Number of competitions per year	4.89	6.35

### Measures

Before completing the scales, participants answered a sociodemographic and sport participation questionnaire developed for this study. This section collected information on age, sex, educational level, family structure, parental participation in physical activity or sport (this combined item was used only for descriptive purposes to identify whether parental figures engaged in either physical activity or sport; it was not used to define participants' eligibility or included in the inferential analyses), sport practiced, age at which participants began practicing sport, weekly training hours, and number of competitions per year. The most frequently reported sports were American football (29%), swimming (15%), soccer (10%), and taekwondo (8%). The remaining participants practiced a variety of individual and team sports.

Two instruments were developed based on the literature and on preliminary qualitative studies conducted with athletes using Natural Semantic Networks (36). The defining words obtained for the stimuli family beliefs about the sport potential of sons and daughters (37) and parental support in sport (38) were used to generate the items. An initial pool of 30 items was developed for each instrument following the criteria established by Morales et al. (39) for constructing Likert-type items, including wording, selection and number of response options, and item order. The Parental Beliefs about Sport Potential Scale (ECPD, Spanish acronym) used five response options ranging from 1 (totally disagree) to 5 (totally agree), whereas the Parental Support in Sport Scale (EAPD, Spanish acronym) used five response options ranging from 1 (never) to 5 (always). The factor structures were not specified *a priori* but were identified through the exploratory factor analyses conducted in the present study. Following these analyses, 22 items were retained for each instrument. The ECPD yielded four factors: Perception of Sport Potential, Parental Expectations, Barriers to Sport Success, and Expected Returns from Sport. The EAPD yielded three factors: Emotional Support and Understanding, Material Resource Support, and Parental Disinterest.

### Procedure

The instruments were administered both in person and online. Two collaborators, recent graduates from the psychology program, were trained in the use of quantitative instruments and data collection. In-person data collection was conducted at secondary, upper-secondary, and higher education institutions in eastern Mexico City. Authorization was first requested from institutional administrators and coaches. Once access was granted, coaches and athletes from the institutional sport teams were contacted and asked to assist with the distribution of the questionnaire among athletes. Those who met the inclusion criteria were invited to participate.

For online data collection, the invitation and questionnaire link were distributed through social media and groups of athletes from eastern Mexico City. The invitation described the inclusion criteria, and interested athletes accessed the questionnaire through Google Forms. In both administration formats, participants provided informed consent before answering the scales, and anonymity, confidentiality, and the protected use of personal data were emphasized. The online questionnaire remained available for one and a half months. Data collection was conducted during the second half of 2025.

The study was approved by the Ethics Committee of the Facultad de Estudios Superiores Zaragoza (UNAM). Adult participants provided written informed consent before answering the instruments. Participants under 18 years of age provided written assent, while their parents or legal guardians were informed about the study through the coaches and could decline or withdraw their child's participation. This procedure was followed for both in-person and online administration. Anonymity, confidentiality, and the protected use of personal data were emphasized in all cases.

### Statistical analysis

Data were analyzed using Jamovi v. 2.6. Descriptive statistics, including means, standard deviations, skewness, and kurtosis, were examined. To obtain validity evidence based on the internal structure of the instruments, exploratory factor analyses were conducted using principal axis factoring with Oblimin rotation. The number of factors was determined through parallel analysis, and factor loadings greater than.30 were retained. Sampling adequacy was evaluated using the Kaiser–Meyer–Olkin index and Bartlett's test of sphericity. Internal consistency was estimated using McDonald's omega coefficient. Pearson bivariate correlations were calculated to examine the associations between the factors of both instruments. Stepwise linear regression analyses were subsequently conducted to identify predictors of the parental support dimensions. Finally, independent-samples Student's *t* tests were used to compare factor scores according to age group (12–18 and 19–25 years) and sex. Statistical significance was set at *p* < 0.05.

## Results

To obtain validity evidence for the Parental Beliefs about Sport Potential Scale (ECPD, Spanish acronym), an exploratory factor analysis with Oblimin rotation was conducted to identify the dimensional structure of the scale. As a previous step, the correlation structure of the data set was examined using the Bartlett test of sphericity, which yielded *χ*² = 7,026 (231 df, *p* < 0.001), and an overall Kaiser-Meyer-Olkin (KMO) value of.919. These results indicate that the variables shared sufficient variance. The distribution of skewness and kurtosis was also examined. The results showed a mesokurtic distribution, with a maximum skewness value of 1.690 and a kurtosis value of 1.701. Based on these results, dimensionality reduction was performed through factor analysis of the data (Costello, 2005). Factor loadings greater than.30 were considered, as they indicate an acceptable relationship between the item and the dimension. The Parental Beliefs about Sport Potential Scale was composed of 22 items distributed across four factors: F1 Perception of Sport Potential, F2 Parental Expectations, F3 Barriers to Sport Success, and F4 Expected Returns from Sport. The total explained variance was 68%, and the total McDonald omega coefficient was.865. The percentages of explained variance and internal consistency values for each factor were as follows: F1 variance = 27.83%, *ω* = 0.909; F2 variance = 17.96%, *ω* = 0.887; F3 variance = 13.46%, *ω* = 0.872; and F4 variance = 8.78%, *ω* = 0.704. The descriptive data of the scale and the factor loadings obtained from the exploratory analysis are shown in Table 2.

**Table 2:** Descriptive data of the parental beliefs about sport potential scale (ECPD, Spanish acronym) and factor loadings obtained from the exploratory analysis.

Items	Descriptive data				Factor loadings			
	Skewness	Kurtosis	M	SD	F1	F2	F3	F4
CP01	−0.667	−0.099	3.80	1.09	**0** **.** **917**	0.048	0.026	−0.055
CP02	−1.034	0.7315	3.97	1.05	**0**.**870**	−0.041	0.044	0.028
CP03	−0.557	−0.240	3.68	1.12	**0**.**868**	0.021	0.059	−0.063
CP04	−1.274	1.4570	4.13	1.01	**0**.**851**	−0.036	−0.032	0.065
CP05	−0.810	0.073	3.87	1.08	**0**.**814**	0.002	−0.084	−0.038
CP06	−1.139	0.974	4.05	1.02	**0**.**788**	−0.048	−0.088	0.076
CP07	−0.581	−0.208	3.74	1.08	**0**.**787**	0.034	0.051	−0.007
CP08	−1.452	1.734	4.22	1.03	**0**.**757**	−0.095	−0.019	0.135
CP09	−0.117	−0.898	3.19	1.28	**0**.**612**	0.193	0.062	−0.012
CP10	1.475	1.574	1.72	1.03	−0.047	**0**.**841**	0.083	−0.010
CP11	1.092	0.653	1.91	1.06	0.131	**0**.**805**	−0.007	0.013
CP12	0.736	−0.416	2.15	1.20	0.156	**0**.**803**	−0.059	0.022
CP13	1.060	0.041	1.94	1.19	−0.130	**0**.**785**	−0.027	−0.059
CP14	1.304	0.810	1.76	1.08	−0.128	**0**.**781**	0.098	0.019
CP15	1.565	1.701	1.68	1.07	0.005	**0**.**740**	0.008	0.051
CP16	0.690	−0.703	2.15	1.25	0.064	−0.116	**0**.**897**	0.023
CP17	0.978	0.125	1.92	1.08	0.010	0.014	**0**.**853**	−0.062
CP18	1.053	0.470	1.87	1.04	−0.048	0.069	**0**.**826**	−0.010
CP19	1.030	0.088	1.94	1.15	−0.023	0.128	**0**.**779**	0.066
CP20	−0.963	0.572	3.94	1.04	−0.055	0.009	0.013	**0**.**827**
CP21	−0.993	0.587	3.96	1.04	−0.060	−0.010	0.011	**0**.**794**
CP22	−0.912	0.430	3.82	1.07	0.210	0.040	−0.025	**0**.**709**

Note: Bold values indicate the primary factor loading of each item.

As with the previous scale, analyses were conducted to obtain validity evidence for the Parental Support in Sport Scale (EAPD, Spanish acronym), through exploratory factor analysis and internal consistency analysis. The results of the Bartlett test of sphericity showed adequate correlation among the data, with *χ*² = 9,778 (231 df, *p* < 0.001), and an overall Kaiser-Meyer-Olkin (KMO) value of.950. Indicators of a mesokurtic structure were also obtained, with skewness values not exceeding 1.798 and kurtosis values not exceeding 2.533.

After conducting the dimensionality reduction analysis, 22 items distributed across three factors were obtained: F1 Emotional Support and Understanding, F2 Material Resource Support, and F3 Parental Disinterest. The statistical results showed adequate precision and usefulness of the instrument, with a total explained variance of 71.3% and a total McDonald omega coefficient of.906, indicating stability of the scale. Values above.70 are considered acceptable (Colorado et al., 2024). The explained variance and internal consistency values for each factor were as follows: F1 variance = 31.7%, *ω* = 0.907; F2 variance = 21.9%, *ω* = 0.944; and F3 variance = 17.7%, *ω* = 0.837. The descriptive data of the items and the factor loadings obtained from the exploratory analysis are shown in Table 3.

**Table 3:** Descriptive data of the items and factor loadings of the parental support in sport scale (EAPD, Spanish acronym).

Items	Descriptive data				Factor loadings		
	Skewness	Kurtosis	M	SD	F1	F2	F3
AP01	−0.871	−0.373	3.86	1.27	**0** **.** **893**	−0.022	0.065
AP02	−0.808	−0.477	3.81	1.28	**0**.**883**	−0.033	0.017
AP03	−0.908	−0.208	3.90	1.23	**0**.**864**	−0.070	0.056
AP04	−0.406	−1.244	3.32	1.49	**0**.**814**	−0.016	0.146
AP05	−0.550	−0.971	3.59	1.38	**0**.**792**	0.059	0.018
AP06	−1.147	0.150	4.06	1.24	**0**.**761**	0.047	−0.039
AP07	−0.710	−0.362	3.88	1.09	**0**.**753**	0.013	−0.168
AP08	−0.745	−0.267	3.92	1.09	**0**.**688**	0.024	−0.219
AP09	−0.833	−0.311	3.90	1.19	**0**.**681**	0.080	−0.080
AP10	−0.911	0.030	4.04	1.06	**0**.**665**	0.079	−0.202
AP11	−0.635	−0.805	3.67	1.33	**0**.**606**	0.214	−0.079
AP12	−0.491	−0.988	3.55	1.36	−0.036	**0**.**917**	0.019
AP13	−0.628	−0.900	3.66	1.39	−0.041	**0**.**913**	−0.022
AP14	−0.949	−0.246	3.92	1.28	−0.001	**0**.**899**	−0.001
AP15	−0.807	−0.671	3.79	1.38	−0.018	**0**.**891**	0.024
AP16	−0.323	−1.105	3.37	1.37	0.024	**0**.**872**	0.022
AP17	−0.775	−0.523	3.80	1.28	0.229	**0**.**710**	−0.069
AP18	1.785	2.533	1.58	0.98	−0.024	0.034	**0**.**915**
AP19	1.798	2.425	1.60	1.04	0.056	0.010	**0**.**904**
AP20	1.567	1.339	1.69	1.13	−0.115	−0.001	**0**.**824**
AP21	1.136	0.147	1.95	1.25	0.009	−0.080	**0**.**780**
AP22	0.931	−0.238	2.04	1.23	−0.005	−0.013	**0**.**729**

Note: Bold values indicate the primary factor loading of each item.

Once the validity evidence results for both scales had been obtained, a parametric correlation analysis was conducted between the factors of the Parental Beliefs about Sport Potential Scale (ECPD, Spanish acronym) and the Parental Support in Sport Scale (EAPD, Spanish acronym). The Pearson bivariate correlation coefficient was used. The correlations indicated that parental beliefs regarding the perception of sport potential were associated with Emotional Support and Understanding and with Material Resource Support, and were negatively associated with Parental Disinterest. In contrast, Parental Expectations were associated with Parental Disinterest and negatively associated with Emotional Support and Understanding. Beliefs about Barriers to Sport Success were associated with Parental Disinterest and negatively associated with Emotional Support and Understanding and Material Resource Support. Finally, Expected Returns from Sport were associated with Emotional Support and Understanding, Material Resource Support, and Parental Disinterest. The statistical data obtained from the correlation analysis are presented in Table 4.

**Table 4:** Pearson r correlation coefficients between the factors of the parental beliefs about sport potential scale and the parental support in sport scale (EAPD, Spanish acronym).

Factors	1	2	3	4	5	6	7
1. Perception of sport potential	—-						
2. Parental expectations	−0.029	—					
3. Barriers to sport success	−0.030	0.559***	—				
4. Expected returns from sport	0.434***	0.019	−0.044	—			
5. Emotional support and understanding	0.667***	−0.121**	−0.182**	0.322***	—		
6. Material resource support	0.439***	−0.069	−0.118**	0.271***	0.558***	—	
7. Parental disinterest	−0.522**	0.279**	0.315***	−0.282**	−0.648**	−0.404**	—

* *p* < 0.05.

** *p* < 0.01.

*** *p* < 0.001.

The explanatory models, developed through linear regression analysis, indicated that Emotional Support and Understanding had an R² = 0.472 and was predicted by the variables of Perception of Sport Potential (*β* = 0.662, *p* < 0.001) and Barriers to Sport Success (*β* = -0.162, *p* < 0.001). The Material Resource Support factor had an R² = 0.211, with Perception of Sport Potential (*β* = 0.394, *p* < 0.001), Barriers to Sport Success (*β* = -0.101, *p* < 0.05), and Expected Returns from Sport (*β* = 0.095, *p* < 0.05) as predictor variables. Finally, Parental Disinterest had an R² = 0.375 and was predicted by Perception of Sport Potential (*β* = -0.511, *p* < 0.001), Parental Expectations (*β* = 0.141, *p* < 0.001), and Barriers to Sport Success (*β* = 0.221, *p* < 0.001).

Finally, a mean difference analysis between two groups was conducted using the Student t test. G1 corresponded to participants between 12 and 18 years of age, and G2 corresponded to those between 19 and 25 years of age. The results showed significant differences in the variables of the Parental Support in Sport Scale. G1 perceived higher Emotional Support and Understanding (t = 3.255, *p* < 0.01, M_G1 = 44.92, SD = 9.21; M_G2 = 41.11, SD = 11.36) and higher Material Resource Support (t = 7.112, *p* < 0.01, M_G1 = 26.12, SD = 5.05; M_G2 = 20.94, SD = 7.21). Significant differences were also found in the factors of the Parental Beliefs about Sport Potential Scale: Perception of Sport Potential (t = 2.760, *p* < 0.01, M_G1 = 36.46, SD = 6.65; M_G2 = 34.14, SD = 8.15) and Parental Expectations (t = 2.346, *p* < 0.05, M_G1 = 12.20, SD = 5.34; M_G2 = 10.87, SD = 5.27). In both cases, G1 obtained higher scores than G2.

The mean difference analysis between two groups using the Student t test, according to sex as the grouping variable, showed significant differences in the Parental Support in Sport Scale only in the Material Resource Support factor (t = −2.720, *p* < 0.01, M_men = 21.36, SD = 7.26; M_women = 23.09, SD = 6.82), with women perceiving higher support. In the Parental Beliefs about Sport Potential Scale, significant differences were found in the Parental Expectations factor (t = 2.430, *p* < 0.05, M_men = 11.65, SD = 5.46; M_women = 10.49, SD = 5.03), with men perceiving higher parental sport expectations. In the Barriers to Sport Success factor (t = 4.653, *p* < 0.001, M_men = 8.55, SD = 4.14; M_women = 6.97, SD = 3.19), men perceived more of these parental behaviors. Finally, in the Expected Returns from Sport factor (t = 2.026, *p* < 0.05, M_men = 11.92, SD = 2.51; M_women = 11.46, SD = 2.48), men obtained higher scores, indicating that sport was more often considered to provide a return on what had been invested.

## Discussion

The study of parental support and parental beliefs is relevant because of the impact that both processes have on the sport development of athletes from early ages. Previous studies have documented the influence of parental support on physical activity and sport (7, 16, 19). However, despite its importance, parental beliefs about the sport potential of sons and daughters have been less explored in scientific research.

In this regard, the present study provided validity evidence based on the internal structure of the two scales used in the research. The Parental Beliefs about Sport Potential Scale (ECPD, Spanish acronym) was composed of four factors: Perception of Sport Potential, which evaluates the parental belief that sons and daughters have the skills, potential, and possibilities for successful development in sport; Parental Expectations, which evaluates the perceived pressure to win competitions or meet high demands during training; Barriers to Sport Success, which refers to parental beliefs associated with factors that may interfere with the sport trajectory, such as school, affective relationships, or other social activities; and Expected Returns from Sport, which refers to the expectation that sport practice may generate future benefits, whether economic, health-related, or related to the prevention of risk behaviors. Morales-Castillo (22) indicated that parental perceptions of the abilities of sons and daughters constitute expectations that connect different domains of performance and are influenced by parental experiences and attributes. Nevertheless, scientific evidence on this topic remains limited, particularly regarding the development of psychometric instruments for its assessment.

Regarding the Parental Support in Sport Scale (EAPD, Spanish acronym), this scale also showed adequate psychometric properties (40, 41). The scale was composed of three factors: Emotional Support and Understanding, with items related to expressions of encouragement, interest in sport practice, and communication with parental figures; Material Resource Support, which refers to the economic and logistical support needed for sport practice, including transportation, food, equipment, and additional expenses; and Parental Disinterest, which represents the absence or low level of parental involvement, expressed through little time dedicated, lack of accompaniment, and limited interest in the sport practiced by sons and daughters. Parental support is a relevant topic in the literature, where instruments such as the Parental Involvement in Sport Scale [EIPD, Spanish acronym (33)] have been identified. This scale includes three factors: Directive Behavior, Support and Understanding, and Active Involvement. These factors present contents close to those identified in the scale validated in the present study. Likewise, the Parental Support Scale for Sport Training in Children [PSCSTS (35)] evaluates dimensions such as policy support, communication support, family support, intrinsic motivation, school support, media support, and training quality support, which confirms the relevance of studying parental support as a multidimensional construct in the sport context.

The results obtained from the correlational analysis indicated an association between parental beliefs regarding the perception of sport potential and both Emotional Support and Understanding and Material Resource Support. According to Hernández et al. (13), parental opinions about the abilities of sons and daughters influence the way they perceive their own skills. Likewise, parental support has been identified as a main driver of intentions to continue participating in sport (8). Parental beliefs are influenced by parental experiences and attributes (22). In this sense, family sport heritage represents an important element for continuity in sport (20). In the present study, a higher proportion of parents did not engage in physical activity or sport compared with those who did, which could have implications for the sport development and formation of their sons and daughters.

A negative association was also found between parental beliefs regarding the perception of sport potential and Parental Disinterest. Hernández et al. (13) reported that, although most parents expect their sons and daughters to continue participating in sport, particularly because of its health benefits, these expectations are not always related to performance or competitive goals. Parental disinterest could affect sport performance, as Teixeira et al. (19) reported that when athletes perceive low parental involvement, performance and commitment to sport activities tend to decrease.

Although parental support is a key element in sport, participants aged 12–18 years perceived greater Emotional Support and Understanding and Material Resource Support than those aged 19–25 years. This difference is consistent with developmental changes in parental roles throughout the sport trajectory. During the early stages of participation, parents commonly manage access to sport, training schedules, transportation, and financial costs. Their direct involvement generally decreases during late adolescence as athletes seek greater autonomy, while in emerging adulthood parental involvement tends to become more indirect and support is gradually reduced as self-sufficiency develops (42). Therefore, the lower scores among older participants may reflect a change in the form and visibility of parental support, particularly tangible support, rather than its complete absence.

Younger participants also perceived higher Parental Expectations and greater Perception of Sport Potential. These findings may be understood within the same developmental process. When parents participate more directly in decisions regarding training and competition, their evaluations of sport ability and their expectations may be more evident to athletes. Hernández et al. (13) indicated that parental beliefs about the abilities of sons and daughters influence how athletes evaluate their own skills. As parental involvement becomes less direct during early adulthood, these beliefs and expectations may also become less salient to athletes. Nevertheless, because the present study was cross-sectional and only collected athletes' perceptions, these differences cannot be interpreted as developmental changes in parental beliefs.

Regarding sex, women perceived greater Material Resource Support, whereas men reported higher Parental Expectations, Barriers to Sport Success, and Expected Returns from Sport. The higher Parental Expectations perceived by men are consistent with the findings of Amado et al. (43), who reported that male athletes perceived greater parental pressure than female athletes. In contrast, the greater Material Resource Support perceived by women could be related to the additional economic, logistical, and access barriers that female athletes may encounter in sport contexts (29). The higher expectations and anticipated returns reported by men may also reflect gendered meanings that associate some sports and sport achievement more strongly with masculine identity (28). However, these interpretations should be considered cautiously because the study did not directly assess gender stereotypes or collect reports from parents.

The present study provides evidence on the importance of the family environment, particularly parental beliefs and parental support, in relation to the sport potential of athletes. The findings suggest that parental influence may affect continuity in sport participation, as well as sport dropout. Support, communication, involvement, and positive beliefs are relevant to motivation and the achievement of sport goals. Therefore, programs that promote parental participation in sport are recommended, not only during the first stages of sport initiation, but also throughout the continuity of the athletic trajectory. Parents should provide direct strategies, such as taking sons and daughters to places where they can be physically active, as well as indirect strategies, such as encouraging them and serving as role models, since both are important for the sport life of a person.

The present study has several limitations. First, the use of non-probability sampling and the recruitment of participants exclusively from eastern Mexico City limit the generalizability of the findings to other populations and geographical contexts. Second, the cross-sectional design does not allow causal relationships or developmental changes associated with age to be established. Third, the study relied exclusively on athletes' perceptions and did not include reports from their parents. The broad age range and the inclusion of participants from different individual and team sports may also have introduced variability in the ways parental beliefs and support were perceived.

Future studies should include the responses of parents, broader samples, and cross-cultural analyses to examine the impact of parental figures in both individual and team sports.

## Data Availability

The raw data supporting the conclusions of this article will be made available by the authors, without undue reservation.
